# Complete Genome Sequence of Klebsiella pneumoniae Podophage Pylas

**DOI:** 10.1128/MRA.01287-19

**Published:** 2019-11-14

**Authors:** Jeffery E. Powell, Lauren Lessor, Chandler O’Leary, Jason Gill, Mei Liu

**Affiliations:** a Center for Phage Technology, Texas A&M University, College Station, Texas, USA; DOE Joint Genome Institute

## Abstract

Carbapenemase-producing Klebsiella pneumoniae is an important opportunistic pathogen due to its drug resistance. This study reports on the isolation and characterization of a podophage, named Pylas, infecting this bacterium. The complete genome of phage Pylas is described, and it is distantly related to the well-studied phage N4.

## ANNOUNCEMENT

Multidrug-resistant Klebsiella pneumoniae poses an urgent threat to public health due to its ability to infect patients with a compromised immune system (1, 2). K. pneumoniae strains producing carbapenemases are resistant to a broad range of antibiotics and can cause infections leading to high mortality rates (3). Phages infecting K. pneumoniae may be used in new therapies for treating this pathogen.

Phage Pylas was isolated from wastewater collected in College Station, TX, in 2015 against a carbapenemase-producing K. pneumoniae isolate. Host bacteria were cultured on tryptic soy broth or agar (Difco) at 37°C with aeration. Phages were cultured and propagated using the soft-agar overlay method (4). The phage was identified as a podophage using negative-stain transmission electron microscopy performed at the Texas A&M University Microscopy and Imaging Center as described previously (5). Phage genomic DNA was prepared using a modified Promega Wizard DNA cleanup kit protocol (5). Pooled indexed DNA libraries were prepared using the Illumina TruSeq Nano LT kit, and the sequence was obtained with the Illumina MiSeq platform using the MiSeq V2 500-cycle reagent kit following the manufacturer’s instructions, producing 773,101 paired-end 250-bp reads for the index containing the phage Pylas genome. FastQC 0.11.5 (https://www.bioinformatics.babraham.ac.uk/projects/fastqc/) was used to quality control the reads. The reads were trimmed with FastX-Toolkit 0.0.14 (http://hannonlab.cshl.edu/fastx_toolkit/download.html) before being assembled using SPAdes 3.5.0 (6). Contig completion was confirmed with PCR using primers (5′-TTGAGTCTGTTCACGCCAAC-3′, 5′-TACCAACAGTTGACCCAGCA-3′) facing off the ends of the assembled contig and Sanger sequencing of the resulting product, with the contig sequence manually corrected to match the resulting Sanger sequencing read. GLIMMER 3.0 (7) and MetaGeneAnnotator 1.0 (8) were used to predict protein-coding genes with manual verification, and tRNA genes were predicted with ARAGORN 2.36 (9). Rho-independent termination sites were identified via TransTermHP (http://transterm.cbcb.umd.edu/). Sequence similarity searches were done using BLASTp 2.2.28 (10) with a maximum expectation cutoff of 0.001 against the NCBI nonredundant (nr), UniProt Swiss-Prot (11), and TrEMBL databases. InterProScan 5.15-54.0 (12), LipoP (13), and TMHMM 2.0 (14) were used to predict protein function. All analyses were conducted at default settings via the CPT Galaxy (15) and WebApollo (16) interfaces (https://cpt.tamu.edu/galaxy-pub).

Phage Pylas was assembled at 79.6-fold coverage into a unit genome of 70,408 bp (17). The GC content of Pylas is 41%, in contrast to the 57% GC content of its *Klebsiella* host (18). As determined by BLASTp, Pylas shares 30 proteins with Escherichia coli podophage N4 (GenBank accession no. NC_008720) (E value, <10^−3^) (19). These shared proteins are involved in DNA replication, transcription, DNA packaging, and morphogenesis. Similar to N4, the Pylas genome has direct terminal repeats, which were predicted by PhageTerm (17) to be 769 bp long; the Pylas genome is generally syntenic with N4. Pylas is closely related to *Klebsiella* phage KpCHEMY26 (GenBank accession no. MN163281), sharing 94% overall nucleotide identity (E value, 0) as determined by BLASTn against the NCBI nucleotide (nt) database. The predicted lysis cassette of Pylas is composed of a holin-antiholin pair, an embedded inner-outer spanin pair, and a peptidoglycan hydrolase endolysin.

### Data availability.

The genome sequence of phage Pylas was submitted to GenBank under the accession no. MH899585. The associated BioProject, SRA, and BioSample accession numbers are PRJNA222858, SRR8556430, and SAMN10909361, respectively.

## References

[1] Struve C, Krogfelt KA 2004 Pathogenic potential of environmental Klebsiella pneumoniae isolates. Environ Microbiol 6:584–590. doi:10.1111/j.1462-2920.2004.00590.x. 15142246

[2] Holt KE, Wertheim H, Zadoks RN, Baker S, Whitehouse CA, Dance D, Jenney A, Connor TR, Hsu LY, Severin J, Brisse S, Cao H, Wilksch J, Gorrie C, Schultz MB, Edwards DJ, Nguyen KV, Nguyen TV, Dao TT, Mensink M, Minh VL, Nhu NT, Schultsz C, Kuntaman K, Newton PN, Moore CE, Strugnell RA, Thomson NR 2015 Genomic analysis of diversity, population structure, virulence, and antimicrobial resistance in Klebsiella pneumoniae, an urgent threat to public health. Proc Natl Acad Sci U S A 112:E3574–E3581. doi:10.1073/pnas.1501049112. 26100894 PMC4500264

[3] Tumbarello M, Viale P, Viscoli C, Trecarichi EM, Tumietto F, Marchese A, Spanu T, Ambretti S, Ginocchio F, Cristini F, Losito AR, Tedeschi S, Cauda R, Bassetti M 2012 Predictors of mortality in bloodstream infections caused by Klebsiella pneumoniae carbapenemase-producing K. pneumoniae: importance of combination therapy. Clin Infect Dis 55:943–950. doi:10.1093/cid/cis588. 22752516

[4] Adams MK 1959 Bacteriophages. Interscience Publishers, Inc, New York, NY.

[5] Gill JJ, Berry JD, Russell WK, Lessor L, Escobar-Garcia DA, Hernandez D, Kane A, Keene J, Maddox M, Martin R, Mohan S, Thorn AM, Russell DH, Young R 2012 The Caulobacter crescentus phage phiCbK: genomics of a canonical phage. BMC Genomics 13:542. doi:10.1186/1471-2164-13-542. 23050599 PMC3556154

[6] Bankevich A, Nurk S, Antipov D, Gurevich AA, Dvorkin M, Kulikov AS, Lesin VM, Nikolenko SI, Pham S, Prjibelski AD, Pyshkin AV, Sirotkin AV, Vyahhi N, Tesler G, Alekseyev MA, Pevzner PA 2012 SPAdes: a new genome assembly algorithm and its applications to single-cell sequencing. J Comput Biol 19:455–477. doi:10.1089/cmb.2012.0021. 22506599 PMC3342519

[7] Delcher AL, Harmon D, Kasif S, White O, Salzberg SL 1999 Improved microbial gene identification with GLIMMER. Nucleic Acids Res 27:4636–4641. doi:10.1093/nar/27.23.4636. 10556321 PMC148753

[8] Noguchi H, Taniguchi T, Itoh T 2008 MetaGeneAnnotator: detecting species-specific patterns of ribosomal binding site for precise gene prediction in anonymous prokaryotic and phage genomes. DNA Res 15:387–396. doi:10.1093/dnares/dsn027. 18940874 PMC2608843

[9] Laslett D, Canback B 2004 ARAGORN, a program to detect tRNA genes and tmRNA genes in nucleotide sequences. Nucleic Acids Res 32:11–16. doi:10.1093/nar/gkh152. 14704338 PMC373265

[10] Camacho C, Coulouris G, Avagyan V, Ma N, Papadopoulos J, Bealer K, Madden TL 2009 BLAST+: architecture and applications. BMC Bioinformatics 10:421. doi:10.1186/1471-2105-10-421. 20003500 PMC2803857

[11] The UniProt Consortium. 2018 UniProt: the universal protein knowledgebase. Nucleic Acids Res 46:2699. doi:10.1093/nar/gky092. 29425356 PMC5861450

[12] Jones P, Binns D, Chang HY, Fraser M, Li W, McAnulla C, McWilliam H, Maslen J, Mitchell A, Nuka G, Pesseat S, Quinn AF, Sangrador-Vegas A, Scheremetjew M, Yong SY, Lopez R, Hunter S 2014 InterProScan 5: genome-scale protein function classification. Bioinformatics 30:1236–1240. doi:10.1093/bioinformatics/btu031. 24451626 PMC3998142

[13] Juncker AS, Willenbrock H, Von Heijne G, Brunak S, Nielsen H, Krogh A 2003 Prediction of lipoprotein signal peptides in Gram-negative bacteria. Protein Sci 12:1652–1662. doi:10.1110/ps.0303703. 12876315 PMC2323952

[14] Krogh A, Larsson B, von Heijne G, Sonnhammer EL 2001 Predicting transmembrane protein topology with a hidden Markov model: application to complete genomes. J Mol Biol 305:567–580. doi:10.1006/jmbi.2000.4315. 11152613

[15] Cock PJ, Gruning BA, Paszkiewicz K, Pritchard L 2013 Galaxy tools and workflows for sequence analysis with applications in molecular plant pathology. PeerJ 1:e167. doi:10.7717/peerj.167. 24109552 PMC3792188

[16] Lee E, Helt GA, Reese JT, Munoz-Torres MC, Childers CP, Buels RM, Stein L, Holmes IH, Elsik CG, Lewis SE 2013 Web Apollo: a Web-based genomic annotation editing platform. Genome Biol 14:R93. doi:10.1186/gb-2013-14-8-r93. 24000942 PMC4053811

[17] Garneau JR, Depardieu F, Fortier LC, Bikard D, Monot M 2017 PhageTerm: a tool for fast and accurate determination of phage termini and packaging mechanism using next-generation sequencing data. Sci Rep 7:8292. doi:10.1038/s41598-017-07910-5. 28811656 PMC5557969

[18] Hua X, Chen Q, Li X, Feng Y, Ruan Z, Yu Y 2014 Complete genome sequence of Klebsiella pneumoniae sequence type 17, a multidrug-resistant strain isolated during tigecycline treatment. Genome Announc 2:e01337-14. doi:10.1128/genomeA.01337-14. 25540349 PMC4276827

[19] Ohmori H, Haynes LL, Rothman-Denes LB 1988 Structure of the ends of the coliphage N4 genome. J Mol Biol 202:1–10. doi:10.1016/0022-2836(88)90512-8. 3172206

